# Linguistic and Cognitive Abilities in Children with Dyslexia: A Comparative Analysis

**DOI:** 10.3390/ejihpe15030037

**Published:** 2025-03-18

**Authors:** Miguel López-Zamora, Nadia Porcar-Gozalbo, Isabel López-Chicheri García, Alejandro Cano-Villagrasa

**Affiliations:** 1 Department of Developmental and Educational Psychology, University of Malaga, 29010 Malaga, Spain; 2 Faculty of Health Sciences, Valencian International University (VIU), 46002 Valencia, Spain; nadia.porcar@professor.universidadviu.com (N.P.-G.); alejandro.cano.v@professor.universidadviu.com (A.C.-V.); 3 UCAM, Universidad Católica de Murcia, 30107 Murcia, Spain; ilchicheri@ucam.edu

**Keywords:** dyslexia, childhood, language, cognition, executive functions

## Abstract

Introduction: Dyslexia is a prevalent learning disorder that significantly affects the child population. It is often accompanied by deficits in language processes, cognition, and executive functioning, all of which are crucial for reading development. Children with dyslexia frequently exhibit difficulties in phonological processing, semantics, morphosyntax, and also in cognitive areas such as working memory, inhibition, planning, and attention. Objective: The primary objective of this study was to compare the linguistic, cognitive, and executive functioning abilities between children diagnosed with dyslexia and those with typical reading development. Methodology: A total of 120 children were selected and divided into two groups: the G-DYSLEXIA group (*n* = 60), consisting of children diagnosed with dyslexia, and the G-CONTROL group (*n* = 60), with typical reading development. Language, cognition, and executive functions were assessed using standardized tests: CELF-5, WISC-V, and ENFEN. Statistical analyses included descriptive statistics, independent sample *t*-tests, and Chi-square tests to compare the performance between these two groups. Results: The study revealed significant differences between the two groups in all dimensions assessed. Specifically, children with dyslexia showed markedly lower performance in linguistic, cognitive, and executive functioning measures compared with their peers with typical development. Conclusion: Children with dyslexia present a distinct clinical profile characterized by significant difficulties in language processing, cognition, and executive functions. These challenges interfere with their reading acquisition and academic performance, limiting their integration into educational environments and impacting their overall quality of life.

## 1. Introduction

The process of learning to read involves multiple biological, cognitive, linguistic, emotional, environmental, socioeconomic, and pedagogical factors (O’Brien & Yeatman, 2021). These factors not only individually affect the acquisition of literacy skills but also interact with each other, making the study of typical reading development highly complex. While most children learn to read without difficulty, a percentage of the population faces significant challenges in this process (Park & Lombardino, 2013). It is estimated that between 30% and 40% of children in early school years experience reading-related difficulties (Sparks et al., 2006).

Literacy problems have a substantial impact on academic performance. Recent studies have shown that more than 15% of children aged 10 to 12 exhibit some form of delay in reading acquisition (Olusanya et al., 2023; Yang et al., 2022). Many of these children remain undiagnosed or do not receive appropriate intervention, contributing to a school dropout rate of approximately 8% among children and adolescents aged 4 to 17 (de Bree et al., 2022; Price et al., 2022). While the origins of reading difficulties are diverse, current research emphasizes the role of specific learning disorders in literacy challenges (Chan et al., 2023). Among these, dyslexia has become a primary focus of interest in both educational and clinical settings.

Dyslexia is a severe, persistent, and specific difficulty in acquiring and developing reading skills. It is considered unexpected because it occurs independently of general cognitive abilities and academic instruction (American Psychiatric Association, 2013). Its origin is not attributed to sensory impairments but rather to a strong genetic and hereditary component (Adlof & Hogan, 2018). It is estimated that approximately 3% to 7% of the population has dyslexia (Jiménez & Rodríguez, 2008; Wagner et al., 2020; Wolff & Lundberg, 2002). Reading difficulties stem from neuroanatomical changes that affect access, processing, and retrieval of phonological information related to speech sounds (Habib & Giraud, 2013; Luque et al., 2011). Likewise, various studies indicate that neuroimaging techniques conducted between the ages of 3 and 5 have proven to be sensitive and predictive in correlating reading impairments in children (Guttorm et al., 2011; Hämäläinen et al., 2013; Leppänen et al., 2012). Similarly, studies such as that of Torppa et al. (2010) suggest that at early stages of development, certain skills predict reading process impairments, such as working memory, verbal memory, processing speed, vocabulary, or rapid naming, all of which can be measured using standardized and validated assessment protocols in Spanish, such as the PROLEXIA instrument, among others.

Models of reading processing have identified two fundamental pathways for word reading (Coltheart et al., 2001): the sublexical or indirect pathway, which involves breaking down words into letters and converting each letter into a sound before merging them into a verbal form, and the lexical or direct pathway, where familiar words are recognized through a mental dictionary. The lexical pathway allows direct access to a word’s meaning, whereas the sublexical pathway requires phonological decoding.

Within these models, reading automation is a key process that promotes fast and fluent reading (Ardila & Cuetos, 2016). In transparent languages such as Spanish, where grapheme–phoneme conversion rules are more predictable, reading speed has proven to be a particularly useful measure for assessing reading performance. Although children with dyslexia may make relatively few errors, they typically exhibit significant deficits in reading speed, making it a crucial diagnostic metric (Defior & Serrano, 2014). On the other hand, accuracy, which combines speed with the number of correct responses, has been the most commonly used measure to assess reading performance in dyslexia (Cappelli et al., 2018). This same finding could apply to other transparent languages, such as Italian. In the study by Carioti et al. (2022), a cohort of monolingual Italian children was evaluated on rapid naming tasks, indicating that this ability is a universal marker for early detection of dyslexia. Similarly, research by Casani et al. (2022) also emphasizes linguistic processes and dimensions such as morphology and syntax as early indicators for diagnosing dyslexia in children in transparent languages such as Italian. In their study, the authors highlighted that morphosyntactic skills influence writing decoding.

However, reading goes beyond these mechanical processes. It also involves broader cognitive functions, such as interpreting word meanings (Smith-Spark, 2018), storing information (Dorofeeva et al., 2022), and retrieving written content (Snowling & Hulme, 2011), all of which contribute to comprehensive reading comprehension (Bishop & Snowling, 2004). Numerous studies have shown that children with dyslexia experience difficulties not only in expressive and receptive language but also in various cognitive processes such as planning, inhibition, attention, and verbal memory, among others (Bazen et al., 2020). Therefore, executive functions play a fundamental role in the proper acquisition of reading processes, as these higher cognitive skills allow for the efficient regulation, supervision, and coordination of the multiple processes involved in reading.

Executive functions such as working memory (Beneventi et al., 2010), cognitive flexibility, and inhibitory control are crucial for retaining and manipulating information while reading, adapting to new tasks or comprehension challenges, and suppressing inappropriate automatic responses to focus on the relevant content of the text (Reiter et al., 2005). These skills are fundamental not only in educational settings but also in everyday life, enabling individuals to function effectively and efficiently across various situations.

Recent studies have highlighted that children with dyslexia exhibit significant deficits in these executive functions, negatively impacting their reading performance. For instance, limited working memory can hinder information retention while decoding words or constructing the meaning of complex sentences (Smith-Spark & Fisk, 2007). This shortfall in working memory means that essential information might be lost in the process, making it difficult to build a coherent and fluent understanding of the text. Moreover, difficulties in inhibitory control may cause children with dyslexia to struggle with avoiding frequent errors or adjusting reading strategies when encountering unfamiliar words (Booth et al., 2010). This difficulty manifests as a reduced ability to ignore irrelevant distractions, which can lead to comprehension errors and less efficient reading. Inhibitory control is crucial for maintaining focus on reading tasks in the face of distracting stimuli or previously automated inappropriate reading habits. Additionally, planning and organization—key components of executive functions—allow readers to anticipate content, set reading goals, and adjust their pace according to the text’s demands. The absence or weakness of these skills in children with dyslexia not only slows down the reading process but also affects overall reading comprehension. This occurs because cognitive resources are excessively concentrated on word decoding, leaving little capacity for interpreting, analyzing, or integrating the information read (Varvara et al., 2014). The consequences of these difficulties are that the reading process becomes slower and more laborious, requiring greater cognitive effort than for children without dyslexia. This can lead to rapid fatigue during reading tasks and decreased motivation to engage in activities involving extensive or complex reading. Over time, these challenges can impact general academic performance and the child’s self-esteem.

The literature suggests that children with impairments in linguistic competencies are at higher risk of developing dyslexia. Specifically, Bishop and Snowling (2004) argue that when comparing children diagnosed with dyslexia and those with language disorders, both share a phonological deficit, but they differ in the extent to which broader language skills, such as vocabulary and comprehension, are affected. These findings have led most dyslexia studies to focus on the role of phonological skills as causal factors in decoding development (Capin et al., 2022; Lyytinen et al., 2005; Ramus & Szenkovits, 2008). However, evidence suggests that broader language skills may also play a crucial role in decoding processes, particularly in children with more advanced literacy experience (Marshall et al., 2011). Morphological awareness has been identified as a unique predictor of variability in word-reading ability in children over the age of 8 (Casani et al., 2022). For example, Giazitzidou and Padeliadu (2022) found that morphological awareness predicted word-reading variations, even after controlling for age, phonological awareness, and naming speed.

Despite the extensive literature on dyslexia, most research has been conducted on English-speaking populations or has examined phonological processing in isolation. There is a lack of comprehensive studies comparing the linguistic and cognitive performance of Spanish-speaking children with dyslexia to that of typically developing readers. Since Spanish is a transparent orthographic language with more predictable grapheme–phoneme conversion rules, it is crucial to investigate how reading difficulties manifest in this linguistic context and whether the reading speed and accuracy deficits observed in other languages also apply to Spanish. Moreover, few studies have explored the impact of executive functions and other cognitive processes, such as working memory and fluid reasoning, in Spanish-speaking children with dyslexia. Understanding these relationships will not only enable more precise identification of areas of deficit but will also enhance diagnostic and intervention strategies, adapting them to the educational and linguistic needs of Spanish-speaking children with dyslexia.

In addition to linguistic difficulties, individuals with dyslexia also exhibit cognitive deficits, particularly in planning and monitoring, which are higher-order cognitive skills associated with executive functions (Smith-Spark & Gordon, 2022). Although there are various perspectives on executive functioning (Miyake et al., 2000), all theories agree on its role in regulating, organizing, and integrating cognitive processes (Denckla, 1996). According to Baddeley’s model (Baddeley, 2007), the central executive is the control system of working memory. However, the relationship between dyslexia and executive functions remains an open question. Studies such as those by Barbosa et al. (2019) suggest that inefficient phonological processing could negatively impact executive functioning, which may explain the cognitive difficulties observed in children with dyslexia.

This study seeks to address existing gaps in the literature by providing empirical evidence on how dyslexia affects both linguistic and cognitive domains in a language with transparent orthography such as Spanish. Additionally, it aims to contribute to the ongoing discussion regarding the role of executive functions in dyslexia and their relationship with reading performance in Spanish-speaking children.

## 2. Materials and Methods

### 2.1. Participants

A total of 120 participants (58 girls and 62 boys) aged between 6 and 8 years (M = 7.2; SD = 0.8) were selected. The sample was evenly divided into two groups: an experimental group of 60 children diagnosed with dyslexia (G-DYSLEXIA) and a control group of 60 children with typical reading development (G-CONTROL).

The process of diagnosing dyslexia in the participants involved a thorough assessment adhering to the established diagnostic criteria of the *Diagnostic and Statistical Manual of Mental Disorders*, Fifth Edition (DSM-5) (American Psychiatric Association, 2013). This manual is widely recognized and used by clinicians globally to ensure consistency and accuracy in the diagnosis of various psychological disorders, including dyslexia. By following these standards, evaluators can reliably identify specific learning disorders related to reading.

Confirmation of the dyslexia diagnosis was achieved using the PROLEC-R assessment battery (Cuetos et al., 2014). The PROLEC-R battery is specifically designed to evaluate the reading abilities of children and is commonly used in both clinical and educational settings to identify specific learning disorders in reading. This battery not only helps in confirming a diagnosis but also in understanding the specific reading challenges faced by each child, which is crucial for planning effective interventions.

The focus on children aged six and seven is particularly important because dyslexia is usually diagnosed after a child has had at least two years of literacy instruction. This timeframe allows for clearer observation of the child’s learning patterns and difficulties in acquiring basic reading skills, which are less evident without formal education. By this age, children have typically been exposed to systematic reading instruction, providing a substantial basis for evaluating their reading abilities and identifying deviations from typical development.

The strict diagnostic criteria included several key components. Firstly, a documented history of potential difficulties in the reading process helps identify children at risk early on. Secondly, the lack of response to early literacy interventions is a critical indicator that standard educational approaches may not be sufficient for the child’s learning needs, suggesting underlying learning disorders. Lastly, a comprehensive clinical evaluation rules out other potential causes of reading impairments, such as sensory impairments or cognitive deficits, ensuring that the diagnosis of dyslexia is specific and accurate.

Once diagnosed, all participants received psychopedagogical support interventions tailored to each child’s needs and delivered by educational psychologists and language and hearing specialists. These interventions are crucial, as they are designed to address the unique learning challenges faced by children with dyslexia. By providing specialized support in the final stages of Early Childhood Education, these interventions aim to equip children with the necessary skills to succeed in later schooling.

In the Spanish educational system, where formal reading instruction begins in the third year of Early Childhood Education (ages 3–4), early identification and intervention are possible and beneficial. Starting pre-reading skill stimulation at this early stage enables educators to address reading difficulties before they become deeply ingrained, thereby enhancing the overall effectiveness of the interventions and improving the academic trajectory for children with dyslexia. This early start is essential to laying a solid foundation for literacy development and ensuring that all children, including those with learning disorders, have the best possible support from the beginning of their educational journey.

To control for potential confounding variables, age matching was performed between the groups, and statistical tests confirmed no significant differences in age distribution. Additionally, since a notable proportion of participants were born before 35 weeks of gestation, care was taken to ensure a balanced distribution of preterm births between groups, minimizing the potential influence of prematurity on cognitive and linguistic outcomes.

All participants were monolingual Spanish speakers born in Spain. The inclusion criteria for the G-DYSLEXIA group required a formal dyslexia diagnosis, an age between six and eight years, and evidence of expressive language development. Exclusion criteria for both groups included severe sensory impairments (auditory or visual), a diagnosis of intellectual disability, or any severe psychiatric disorder that might interfere with cognitive and linguistic evaluations. While comorbidity with other neurodevelopmental disorders was controlled for, it was not considered an exclusionary criterion in the G-DYSLEXIA group, ensuring a sample representative of dyslexic populations at the population level.

### 2.2. Instruments and Materials

The following instruments were used to assess the linguistic and cognitive abilities in the participants:

CELF-5. Clinical Evaluation of Language Fundamentals—5th Edition (Wiig et al., 2018): This battery assesses the language skills of children and adolescents between the ages of 5 and 15. Its main objective is to detect language and communication disorders through 12 tests, with each subtest containing between 10 and 15 items, including sentence comprehension, linguistic concepts, and morphosyntax. The tests are divided into two parts based on the individual’s age and are grouped into five general indices that describe various aspects of language: comprehension, expression, and linguistic structure. It also includes a questionnaire for parents and teachers that evaluates communicative performance in school and family environments. The psychometric scores, calculated based on our sample, show a Cronbach’s alpha of 0.91, indicating high internal consistency. For this study, variables such as semantics, morphosyntax, pragmatics, and auditory comprehension were used. This battery comprehensively assesses language skills, including sentence comprehension, linguistic concepts, and morphosyntax. Therefore, all its subtests require significant verbal abilities to evaluate various aspects of language such as comprehension, expression, and linguistic structure.

WISC-V. Wechsler Intelligence Scale for Children—5th Edition (Wechsler, 2010): This clinical instrument is used to assess cognitive ability in children aged 6 to 16 years. The WISC-V provides composite scores for cognitive domains such as verbal comprehension, fluid reasoning, and working memory, as well as a full-scale score representing general intellectual ability. The battery consists of 10 primary subtests (ranging from 15 to 20 items each) and 5 supplementary subtests. The reliability analysis, based on our sample, yielded a Cronbach’s alpha of 0.89, confirming high internal consistency. This instrument offers a diverse assessment that includes areas requiring verbal skills and others that do not. The verbal comprehension subtests, which involve understanding and manipulating spoken language, directly depend on linguistic skills. However, other subtests like those assessing fluid reasoning and working memory may not require direct verbal abilities, as they evaluate the capacity to reason and manipulate information in formats that are not exclusively verbal.

ENFEN. Neuropsychological Assessment of Executive Functions in Children (Portellano et al., 2009): This battery is designed to assess executive functions in children aged 6 to 12 years. The ENFEN consists of four subtests: verbal fluency (15 items), trail making (20 items), ring construction (15 items), and resistance to interference (15 items). The internal consistency of the instrument, calculated from our study’s sample, showed a Cronbach’s alpha of 0.87, ensuring high reliability. The ENFEN is widely used in the assessment of children with cognitive difficulties. This battery combines tests that assess both verbal and non-verbal skills. Specifically, the verbal fluency subtest requires children to generate words under certain constraints, making intensive use of linguistic skills. The other subtests, such as trail making, ring construction, and resistance to interference, focus more on assessing cognitive and psychomotor skills, where the linguistic component is minimal or non-essential. These examine how children organize their thoughts and movements efficiently and how they handle distractions.

### 2.3. Procedure

The study was approved by the Ethics Committee of the University of Málaga (UMA) under approval code 120-2023-H. All families of the participants provided informed consent to participate in the study, ensuring compliance with data protection regulations and confidentiality.

Data collection was carried out in two main phases. In the first phase, an initial interview was conducted with the parents or guardians of the participants to collect sociodemographic information, medical and academic history, and other relevant details for the evaluation (such as family history of learning difficulties or comorbidities). During this interview, further details about the study, its objectives, and the evaluation procedure were explained, addressing any questions the families might have had. Following this, the assessment sessions took place. The linguistic and cognitive assessments were administered individually to each participant in a quiet, controlled environment, such as a dedicated room within their school or a designated area in the reference hospital. The evaluation was spread across three sessions to avoid participant fatigue, with each session lasting approximately 90 min. These sessions were scheduled over the course of a week to ensure that the participants could perform optimally in each session. In the first session, linguistic assessments were conducted using the CELF-5. The second session focused on cognitive assessments, where the WISC-V was administered to evaluate general cognitive ability. The third session involved the ENFEN battery to assess executive functions, allowing for a comprehensive analysis of the participants’ cognitive development.

Each session was conducted by trained evaluators (psychologists and speech therapists with experience in administering these tests) to ensure the accurate application of the instruments and the validity of the results. Special care was taken to include breaks during the sessions if the children showed signs of fatigue to maintain the validity of their responses. The study included a total of 120 participants, 62 male (51.6%) and 58 female (48.4%). Of these, 60 participants were diagnosed with dyslexia (32 male and 28 female), while 60 participants did not have a dyslexia diagnosis (30 male and 30 female). In the second phase, the collected data were reviewed and organized into a database for analysis. Quality control procedures were implemented to ensure the accuracy of data entry and to avoid any coding errors. The data were then prepared for comprehensive statistical analysis using the tools selected for this study. In addition, follow-up communication was maintained with the participants through their schools and families, ensuring that no additional factors interfered with their performance during the evaluations. The entire data collection process was carried out over a period of three months.

### 2.4. Design

This is a descriptive, cross-sectional study with an experimental group (G-DYSLEXIA) and a control group (G-CONTROL). The dependent variables were the linguistic and cognitive functions assessed through the standardized instruments mentioned earlier. The independent variable was the diagnosis of dyslexia.

A descriptive statistical analysis of the same variables was then conducted. To compare the two groups, an independent samples *t*-test was performed with a Bonferroni correction applied to control for the risk of Type I errors. The statistical analyses were conducted using SPSS version 26.

## 3. Results

First, various descriptive analyses were conducted, and the homogeneity of the participant sample was verified for each sociodemographic variable. The results of these analyses are reflected in Table 1 below:

### 3.1. Comparison of Linguistic Performance

The difference in scores was calculated using the independent samples *t*-test for variables related to linguistic competencies. The results showed significant differences in all variables related to language. The results of Levene’s test and the *t*-test analysis for language performance are described in Table 2.

The statistical results presented in the table reveal highly significant differences between the G-DYSLEXIA and G-CONTROL groups across all the linguistic skills assessed using an independent samples *t*-test. Firstly, the Levene’s test for equality of variances yielded significance values greater than 0.05 for all variables, confirming that there were no significant differences in the variances between the two groups, thus allowing the assumption of homogeneity of variances in the analysis. In terms of sentence comprehension, the G-DYSLEXIA group had a mean of 6.58 (SD = 1.64) compared with a mean of 10.28 (SD = 2.12) in the G-CONTROL group. The *t*-test analysis showed t(118) = 7.594, with a bilateral significance of *p* < 0.001, confirming a highly significant difference between the groups. The effect size calculated using η^2^ was 0.845, indicating a very large effect. The mean difference between the groups was 3.700 points, with a Cohen’s d value of 1.95, further emphasizing the magnitude of this disparity in sentence comprehension. Regarding linguistic concepts, children with dyslexia scored a mean of 5.68 (SD = 1.89) compared with a mean of 11.26 (SD = 1.96) in the control group. The *t*-test resulted in t(118) = 8.648, *p* < 0.001, again indicating a highly significant difference. The effect size was considerably large (η^2^ = 0.685), and the mean difference between the groups was 5.580 points, with a d value of 2.89, reflecting a substantial difference in the ability to handle linguistic concepts. Morphosyntax also showed significant differences, with a mean of 7.84 (SD = 1.29) in G-DYSLEXIA compared with 14.17 (SD = 2.67) in G-CONTROL. The *t*-test yielded t(118) = 6.484, *p* < 0.001, with an effect size of η^2^ = 0.954, indicating that the variance explained by the group is very high. The mean difference was 6.330 points, and the d value was 3.01, suggesting that morphosyntactic ability is significantly lower in children with dyslexia. For the pragmatic skills profile, the G-DYSLEXIA group had a mean of 3.87 (SD = 1.35), significantly lower than the control group’s mean of 9.46 (SD = 1.35), with t(118) = 4.654, *p* < 0.001. The effect size was notable (η^2^ = 0.659), with a mean difference of 5.590 points and a d value of 4.14, reflecting significant pragmatic difficulties in children with dyslexia. Finally, oral text comprehension also showed significant differences, with a mean of 5.12 (SD = 3.84) in G-DYSLEXIA compared with 10.53 (SD = 1.99) in G-CONTROL. The *t*-test showed t(118) = 7.498, *p* < 0.001, with an effect size of η^2^ = 0.953 and a mean difference of 5.410 points (d = 1.76), indicating a considerable deficit in oral text comprehension in the dyslexic group.

### 3.2. Comparison of Cognitive Performance and Executive Functioning

The difference in scores was calculated using the independent samples *t*-test for variables related to cognitive competencies. The results showed significant differences in all variables related to cognition. The results of the Levene’s test and the *t*-test analysis for cognitive performance are described in Table 3 and Table 4.

The results from the comparative analysis of cognitive processes between the two groups, G-DYSLEXIA and G-CONTROL, using the WISC-V subscales, provide a robust insight into the cognitive disparities between children diagnosed with dyslexia and their typically developing peers. The statistical tests conducted across various cognitive domains—verbal comprehension, visuospatial skills, fluid reasoning, working memory, and processing speed—reveal highly significant differences, underscoring the impact of dyslexia on cognitive function. In the realm of verbal comprehension, G-CONTROL (control group) displayed significantly superior abilities compared with G-DYSLEXIA (dyslexia group). The mean difference in scores was 5.850 points, with a t-value of 10.374, which is highly significant (*p* < 0.001). The effect size, as indicated by η^2^, was 0.801, suggesting a large effect. The Cohen’s delta of 2.65 further confirmed a substantial difference between the groups. This implies that children without dyslexia have a much stronger grasp of language-based tasks such as understanding and processing verbal information, which are crucial for effective communication and academic performance. Similarly, in visuospatial skills, which involve the ability to understand and remember the visual and spatial relations among objects, G-CONTROL exhibited significantly better performance, with a mean difference of 7.430 points. The statistical significance of this disparity was marked by a t-value of 9.515 (*p* < 0.001) and an even larger effect size (η^2^ = 0.841), with a Cohen’s delta of 4.16. These results highlight that children in the control group are better at visualizing and manipulating objects, skills important for subjects like mathematics and sciences. In fluid reasoning, which is the capacity to think logically and solve problems in novel situations, independent of acquired knowledge, G-CONTROL also significantly outperformed G-DYSLEXIA. The mean difference here was 5.540 points, with a t-value of 7.549 (*p* < 0.001), an effect size of 0.675, and a Cohen’s delta of 2.26. Fluid reasoning is critical for the general understanding and handling of abstract concepts, indicating that the control group possesses stronger abilities in adapting to new cognitive challenges. For working memory, which reflects the ability to hold and manipulate information over short periods, G-CONTROL again stood out, with a mean difference of 4.370 points, a t-value of 6.419, and a highly significant *p*-value (< 0.001). The effect size was 0.765, and Cohen’s delta was 2.37. This suggests that children without dyslexia can better manage multiple pieces of information simultaneously, a skill fundamental to many educational and everyday tasks. Lastly, in processing speed, the ability to perform simple cognitive tasks quickly and fluently, G-CONTROL showed superior performance, with a mean difference of 5.620 points, mirrored by the same t-value as fluid reasoning (7.549), a significant *p*-value, an effect size of 0.680, and a Cohen’s delta of 3.37. Faster processing speed in the control group suggests a more efficient cognitive system that can execute tasks more swiftly, beneficial in both academic settings and daily life.

In the analysis of executive functions between the groups G-DYSLEXIA and G-CONTROL evaluated through the ENFEN subscales, statistical tests were conducted to examine differences in interference resistance, trail making, verbal fluency, and ring construction. The results highlighted highly significant disparities between the groups in all evaluated areas, indicating notable differences in executive functions. In interference resistance, G-CONTROL exhibited significantly superior performance compared with G-DYSLEXIA (t(118) = 8.459, *p* < 0.001, η^2^ = 0.856, δ = −2.39), with a mean difference of 7.000 points. Similarly, in trail making, G-CONTROL showed significantly better performance (t(118) = 8.148, *p* < 0.001, η^2^ = 0.652, δ = −1.97), with a mean difference of 5.567 points. In verbal fluency, G-CONTROL significantly outperformed G-DYSLEXIA (t(118) = 7.198, *p* < 0.001, η^2^ = 0.796, δ = −2.21), with a mean difference of 5.667 points. In ring construction, G-CONTROL stood out significantly (t(118) = 6.148, *p* < 0.001, η^2^ = 0.751, δ = −1.87), with a mean difference of 5.000 points. These results underscore the presence of substantial disparities in executive functions between the two groups, highlighting the superior performance of G-CONTROL in all evaluated dimensions of the ENFEN.

## 4. Discussion

The primary objective of this study was to compare the linguistic and cognitive performance of two groups of children: one diagnosed with dyslexia (G-DYSLEXIA) and another with typical reading development (G-CONTROL). The results confirm the initial hypotheses, showing significant deficiencies in all evaluated linguistic and cognitive competencies in children with dyslexia compared with the control group. Below, we discuss these findings in greater depth, exploring the possible underlying causes and their implications in academic and clinical contexts.

The analysis of linguistic competencies reveals that children with dyslexia present significantly lower performance in all evaluated areas: sentence comprehension, linguistic concepts, morphosyntax, pragmatics, and oral text comprehension. It is essential to clarify that, in this study, dyslexia is defined according to the DSM-5 TR criteria, specifically, as a specific learning disorder characterized by difficulties in accurate or fluent word recognition, spelling, and decoding, without including reading comprehension disorder as part of the diagnosis. The differences observed between the groups are not only statistically significant but also clinically relevant, as they affect key areas of language development essential for academic success and daily communication.

One of the main explanations for these deficiencies lies in the altered phonological processing that characterizes reading and writing difficulties (Hulme & Snowling, 2016). Children with dyslexia struggle to segment, identify, and manipulate speech sounds, which directly impacts their ability to process oral and written language. This limitation is crucial in tasks such as sentence comprehension and linguistic concepts, where the quick and precise identification of phonemes and words is essential for proper understanding. Additionally, difficulties in morphosyntax reinforce the idea that dyslexia affects not only word recognition but also the construction and comprehension of complex grammatical structures. Errors in verb conjugation and the construction of complex sentences observed in the G-DYSLEXIA group align with previous studies suggesting that children with dyslexia have difficulties acquiring and automating grammatical rules (Marshall et al., 2011). A possible explanation is that children with dyslexia must allocate more cognitive resources to word decoding, leaving fewer resources available for processing morphosyntactic rules, which compromises their grammatical production and comprehension.

The results in pragmatic skills also revealed significant differences, suggesting that children with dyslexia face difficulties not only at the phonological and grammatical levels but also in the effective use of language in social contexts. Pragmatics, which involves the appropriate use of language in everyday situations, appears to be impaired in these children, limiting their ability to participate autonomously in social activities. This may be partly due to anxiety or frustration stemming from their linguistic difficulties, inhibiting social interaction and affecting their ability to navigate complex communicative situations (Coltheart et al., 2001).

Regarding oral text comprehension, the significant difference observed between the groups highlights how dyslexia affects children’s ability to process and retain orally presented information. Children with dyslexia, facing difficulties in working memory and phonological processing, struggle to follow and understand the flow of an oral text, impairing the construction of a coherent understanding of the message (Snowling et al., 2020). It is important to note that these difficulties do not stem from a primary comprehension deficit but from the phonological challenges inherent in dyslexia, reinforcing the importance of differentiating between dyslexia and reading comprehension disorders in this study.

The results of cognitive and executive function tests also revealed notable differences between the groups across all evaluated areas. Children with dyslexia scored significantly lower in verbal comprehension, fluid reasoning, working memory, and processing speed, highlighting the complexity of dyslexia, which affects not only language but also fundamental cognitive skills essential for learning. These findings align with studies conducted in other transparent languages, such as Italian, where children with dyslexia also exhibit deficits in executive functions, including working memory and cognitive flexibility (Mascheretti et al., 2017). This suggests that orthographic transparency does not eliminate executive function deficits in dyslexia but may influence how these deficits manifest.

Verbal comprehension was one of the most affected areas in children with dyslexia. In examining the cognitive deficits associated with dyslexia, it is crucial to differentiate the root causes of these difficulties. This study specifically attributes the observed deficits in reading and related cognitive processes to the phonological processing challenges inherent in dyslexia as opposed to issues primarily arising from verbal comprehension disorders. This distinction is vital for understanding the specific nature of dyslexia and for tailoring appropriate educational interventions.

Phonological processing—the ability to discern and manipulate sounds in speech—has long been recognized as a core difficulty for those with dyslexia. Individuals with this disorder often struggle with tasks such as rhyming, segmenting sounds from words, and rapidly naming series of random letters or numbers, all of which are critical for effective reading and spelling. This contrasts with a primary verbal comprehension disorder, which would imply broader difficulties in understanding spoken language, extending beyond the decoding issues associated with dyslexia.

The conceptual framework adopted from Hulme and Snowling (2016) supports this interpretation by emphasizing the phonological deficits as central to dyslexia. According to their research, difficulties in phonological processing can impede the development of efficient reading skills, which in turn affects reading fluency and comprehension. By referencing this framework, the study aligns with established theoretical perspectives that advocate for a phonological processing deficit model in dyslexia, reinforcing the specificity of the challenges faced by individuals with this condition.

Integrating this clarification into the discussion section of the study is not just about academic rigor; it also serves a practical purpose. It ensures that the findings are interpreted correctly and that the implications for intervention are appropriate. Misattributing these deficits to a primary verbal comprehension disorder could lead to less targeted, and thus, less effective interventions, potentially focusing on broader language skills rather than the specific phonological skills that individuals with dyslexia need to develop.

Moreover, this focus helps in communicating the findings to educators and clinicians who are involved in designing and implementing educational strategies. Understanding that the core issue lies in phonological processing rather than general verbal comprehension allows for more focused educational approaches. These might include phonics-based reading programs, which have been shown to significantly improve the reading abilities of children with dyslexia by strengthening their phonological processing capabilities.

Therefore, by emphasizing that the deficits observed are linked specifically to the phonological processing challenges associated with dyslexia, the study not only aligns with a well-established body of research but also guides future educational practices and interventions toward more effective and specific methodologies that address the root of the problem. This clarification ensures that both the academic community and practitioners are on the same page regarding the nature of dyslexia and the most effective ways to support those affected by it.

Previous studies have shown that problems in verbal comprehension are related to difficulties in accessing and using mental lexicons as well as applying grammatical rules effectively (Hulme & Snowling, 2016). In this context, children with dyslexia must exert additional cognitive effort to understand and process verbal information, leading to cognitive fatigue and reducing the accuracy and speed of their responses.

Fluid reasoning also showed significant differences, as it involves the ability to solve novel problems, requiring cognitive flexibility and the integration of different types of information. Children with dyslexia seem to struggle in this area due to the additional cognitive load they face when processing written language. This overload limits their ability to allocate sufficient cognitive resources to tasks requiring logic and abstract reasoning (Giazitzidou & Padeliadu, 2022). Working memory, crucial for temporarily holding and manipulating information, was also significantly lower in the children with dyslexia, affecting both their reading ability and overall performance in complex cognitive tasks.

Regarding executive functions, the results revealed substantial differences between the groups in tests of interference resistance, verbal fluency, ring construction, and trail making (Marshall et al., 2011). Executive functions, such as inhibition, attentional control, and planning, are essential for problem solving and managing cognitive demands effectively. Difficulties observed in interference resistance and verbal fluency suggest that children with dyslexia struggle to suppress automatic responses and flexibly switch between tasks, consistent with the literature documenting deficits in inhibition and cognitive flexibility in this population (Barbosa et al., 2019). The consideration of prematurity in studies involving cognitive development is crucial due to its potential impact on neurological development and subsequent cognitive functions. Prematurity has been consistently linked with a variety of developmental challenges, including delays in cognitive and language skills that could confound results when studying conditions such as dyslexia. Recognizing this, the study took meticulous steps to control for the variable of prematurity, ensuring that any conclusions drawn about the cognitive abilities of participants, particularly their executive functions and reading skills, were not unduly influenced by this factor.

In the execution of the study, the researchers ensured an equitable distribution of preterm births between the dyslexic and non-dyslexic groups. This strategy involved a careful selection process where the history of each participant was reviewed and categorized. By matching the number of participants born prematurely in each group, the study aimed to neutralize the impact of prematurity as a confounding variable. This was essential to isolate the effects of dyslexia on cognitive outcomes from those potentially attributable to early birth.

The use of a normal distribution of preterm births across both groups is a statistical approach that further strengthens the study’s design. By ensuring that the sample resembles a normal curve with respect to the timing of birth, researchers mitigate the risk of skewed results that could favor one group over the other. This normal distribution implies that the sample contains a range of preterm to full-term births in proportions that are statistically expected, which helps in generalizing the findings to a broader population.

However, it is important to note that even with such controls in place, prematurity may still exert subtle influences on the developmental trajectories of children. While it was not considered the primary explanatory factor for the observed deficits in this study, prematurity could interact with dyslexia in ways that are not fully understood, potentially affecting the severity or manifestation of dyslexic symptoms. Longitudinal studies and further research could explore these interactions in depth, examining how early birth influences learning and cognitive development over time in the presence of dyslexia.

Thus, by meticulously controlling for prematurity during participant selection, the study provides a clearer, more accurate assessment of the cognitive and executive function deficits attributable to dyslexia, minimizing the potential interference of prematurity as a confounding factor. This approach not only enhances the validity of the study’s conclusions but also contributes to a more nuanced understanding of how different factors influence cognitive development in children with learning disorders.

It is important to note that these executive function deficits are not exclusive to dyslexia but may be related to the additional cognitive effort these children must exert to process language (Snowling et al., 2020). The cognitive overload resulting from language decoding difficulties reduces the resources available for tasks requiring inhibitory control and sustained attention. This reinforces the idea that reading difficulties and executive functions are interrelated, affecting multiple aspects of academic performance and overall development.

From a neurobiological perspective, several studies have identified structural and functional abnormalities in brain areas related to language processing in individuals with dyslexia, such as the angular gyrus and inferior frontal gyrus, which are involved in phonological decoding and information integration (Mascheretti et al., 2017). These neuroanatomical alterations may explain why children with dyslexia struggle with tasks requiring manipulation and recognition of speech sounds, affecting their reading and writing abilities.

Finally, the epigenetic approach highlights how genetic and environmental factors interact to influence the expression of dyslexia, emphasizing that while there is a genetic predisposition, factors such as the quality of the educational environment and family support can modulate the severity and manifestation of symptoms. This is key to understanding why some children with dyslexia respond better to educational and therapeutic interventions, while others face persistent difficulties (López-Resa & Moraleda-Sepúlveda, 2023). Therefore, adopting a holistic approach to dyslexia treatment, integrating educational interventions with emotional and family support, is essential.

## 5. Conclusions

In conclusion, the results of this study confirm that individuals with dyslexia exhibit substantial alterations in language and cognition processes, supporting previously formulated research hypotheses. Significant difficulties were identified in various linguistic skills, consistent with the current scientific literature. These include alterations in phonological coding, naming speed, lexical richness, morphosyntax, pragmatics, and oral comprehension, contributing to an altered linguistic profile in the population with dyslexia. Limitations in phoneme identification and categorical perception and reduced vocabulary suggest difficulties accessing the functional lexicon and executing routes during automatic reading. Additionally, cognitive and executive functioning difficulties were found in the children with dyslexia compared with the children with typical reading performance. The results align with the scientific literature, demonstrating alterations in executive skills such as planning, inhibition, emotional control, working memory, organization, and monitoring. These difficulties, from a neuropsychological perspective, are rooted in structural and functional anomalies in brain areas associated with language processing, affecting phonological decoding and integration. The additional cognitive load when processing written information can explain the alterations in executive functioning such as organization and monitoring.

## Figures and Tables

**Table 1:** Characteristics of the study participants.

	Dyslexia (N, %)	No Dyslexia (N, %)
Gender		
Male	35 (58.3%)	27 (45.0%)
Female	25 (41.7%)	33 (55.0%)
Age		
6 years	20 (33.3%)	22 (36.7%)
7 years	18 (30.0%)	19 (31.7%)
8 years	22 (36.7%)	19 (31.7%)
Years of Treatment		
1 year	33 (55.0%)	0
2 years	15 (25.0%)	0
3 years	12 (20.0%)	0
Comorbidity		
Dysgraphia	18 (30.0%)	0
Dyscalculia	20 (33.3%)	0
Dysgraphia and dyscalculia	22 (36.7%)	0
School Support		
Yes	30 (50.0%)	0
No	30 (50.0%)	0
Weeks of Gestation		
30–35	18 (30.0%)	20 (33.3%)
35–40	20 (33.3%)	20 (33.3%)
More than 40	22 (36.7%)	20 (33.3%)
Apgar		
At risk	16 (26.7%)	18 (30.0%)
Intermediate	22 (36.7%)	21 (35.0%)
Normal	22 (36.7%)	21 (35.0%)

**Table 2:** Results of differences in measures of linguistic competence between G-DYSLEXIA and G-CONTROL.

Language Skills	Groups		*t*-Test for Equality of Means				
	G-DYSLEXIA	G-CONTROL	t	df	Sig. (Bilateral)	Mean Difference	ð (SE)
Sentence Comprehension	M = 6.58 SD = 1.64	M = 10.28 SD = 2.12	7.594	118	<0.001	3.700	1.95 (0.22)
Linguistic Concepts	M = 5.68 SD = 1.89	M = 11.26 SD = 1.96	8.648	118	<0.001	5.580	2.89 (0.26)
Morphosyntax	M = 7.84 SD = 1.29	M = 14.17 SD = 2.67	6.484	118	<0.001	6.330	3.01 (0.26)
Pragmatic Skills Profile	M = 3.87 SD = 1.35	M = 9.46 SD = 1.35	4.654	118	<0.001	5.590	4.14 (0.32)
Oral Text Comprehension	M = 5.12 SD = 3.84	M = 10.53 SD = 1.99	7.498	118	<0.001	5.410	1.76 (0.21)

**Table 3:** Results of differences in measures of cognitive competence between G-DYSLEXIA and G-CONTROL (WISC-V).

Cognitive	Groups		*t*-Test for Equality of Means				
	G-DYSLEXIA	G-CONTROL	t	df	Sig. (Bilateral)	Mean Difference	ð (SE)
Verbal Comprehension	M = 7.11 SD = 1.58	M = 12.96 SD = 2.68	10.374	118	<0.001 *	5.850	2.65 (0.25)
Visuospatial	M = 6.48 SD = 1.52	M = 13.91 SD = 2.01	9.515	118	<0.001 *	7.430	4.16 (0.32)
Fluid Reasoning	M = 6.15 SD = 1.85	M = 11.69 SD = 2.93	7.549	118	<0.001 *	5.540	2.26 (0.23)
Working Memory	M = 5.99 SD = 2.02	M = 10.36 SD = 1.64	6.419	118	<0.001 *	4.370	2.37 (0.24)
Processing Speed	M = 6.09 SD = 1.61	M = 11.71 SD = 1.72	7.549	118	<0.001 *	5.620	3.37 (0.28)

* *p* < 0.05.

**Table 4:** Results of differences in measures of executive functioning between G-DYSLEXIA and G-CONTROL (ENFEN).

Executive Functions	Groups		*t*-Test for Equality of Means				
	G-DYSLEXIA	G-CONTROL	t	df	Sig. (Bilateral)	Mean Difference	ð (SE)
Interference Resistance	M = 5.37 SD = 2.51	M = 12.37 SD = 3.28	8.459	118	<0.001 *	7.000	−2.39 (0.23)
Trail Making	M = 6.50 SD = 2.76	M = 12.07 SD = 2.90	8.148	118	<0.001 *	5.567	−1.97 (0.22)
Verbal Fluency	M = 7.00 SD = 2.80	M = 12.67 SD = 2.29	7.198	118	<0.001 *	5.667	−2.21 (0.23)
Ring Construction	M = 5.97 SD = 2.95	M = 10.97 SD = 2.35	6.148	118	<0.001 *	5.000	−1.87 (0.21)

* *p* < 0.05.

## Data Availability

The data presented in this study are available on request from the corresponding author. The data are not publicly available due to specific ethical and privacy considerations.
